# Effectiveness, Usability, Cost of Nudge-Based SS-RE in older adults with MCI: A HC-Mixed Design Study

**DOI:** 10.1093/geroni/igaf122.2735

**Published:** 2025-12-31

**Authors:** Xiaoyan Zhao, Qiaoqin Wan, Yue Lan

**Affiliations:** Peking University Health Science Center, Beijing, Beijing, China (People’s Republic); Peking University, Beijing, Beijing, China (People’s Republic); Peking University Health Science Center, Beijing, Beijing, China (People’s Republic)

**Keywords:** Exercise, MCI, nudge, Semi-supervised intervention

## Abstract

**Objective:**

This study aimed to evaluate the effect, usability, and cost-effectiveness of the nudges-based semi-supervised resistance exercise (SS-RE) program in older adults with mild cognitive impairment (MCI).

**Methods:**

A non-randomized intervention study with a historical control (HC) group [fully-supervised resistance exercise] was conducted. In the intervention group, participants engaged in the SS-RE program integrated with nudging strategies. Quantitative data on exercise adherence, cognitive function, and physical function were collected and compared between the two groups. Economic evaluation was conducted from the healthcare professional’s perspective. Qualitative interviews assessed participants’ experiences of the nudges.

**Results:**

The nudges-based SS-RE program demonstrated comparable exercise adherence, cognitive function, and physical function to the historical control group, with reduced professional labor costs. Almost all nudges were perceived as beneficial, expect for the precommitment. The qualitative feedback from the participants supported the quantitative findings and provided insights that may explain them. It was noteworthy that some nudges had minimal impacts, and some showed initial promise but influence diminished over time.

**Conclusion:**

The SS-RE program with nudges offered similar health benefits to fully-supervised resistance exercise at lower cost. Future large-scale randomized controlled trials are needed to validate these findings.

